# A Case of Preeclampsia with Uterine Necrosis after Uterine Artery Embolization for Postpartum Hemorrhage

**DOI:** 10.1155/2022/2859766

**Published:** 2022-05-17

**Authors:** Midori Yoshikawa, Takahiro Seyama, Takayuki Iriyama, Seisuke Sayama, Tatsuya Fujii, Masatake Toshimitsu, Moto Nakaya, Ryo Kurokawa, Eisuke Shibata, Takeyuki Watadani, Keiichi Kumasawa, Takeshi Nagamatsu, Kaori Koga, Yutaka Osuga

**Affiliations:** ^1^Department of Obstetrics and Gynecology, Faculty of Medicine, The University of Tokyo, 7-3-1 Hongo, Bunkyo-ku, Tokyo 113-8655, Japan; ^2^Department of Radiology, Faculty of Medicine, The University of Tokyo, 7-3-1 Hongo, Bunkyo-ku, Tokyo 113-8655, Japan

## Abstract

Uterine necrosis is a rare complication in uterine artery embolization (UAE) for postpartum hemorrhage (PPH). Preeclampsia (PE) is a condition characterized with systemic endothelial damage and intravascular volume depletion. Whether a patient with PE is at high risk for uterine necrosis after UAE for PPH has been unknown. A 30-year-old primipara woman was diagnosed with PE based on hypertension and proteinuria during delivery. UAE was performed for PPH after forceps delivery. After UAE, the patient presented with pleural effusion and massive ascites as well as persistent fever unresponsive to antibiotics. Ultrasonography and contrast-enhanced magnetic resonance imaging (MRI) led to the diagnosis of uterine necrosis, for which we performed total laparoscopic hysterectomy. It should be kept in mind that patients with PE associated with massive ascites may be at high risk for uterine necrosis after UAE due to decreased uterine perfusion. Therefore, it is important to pay attention to persistent symptoms such as fever and abdominal pain after UAE to diagnose uterine necrosis.

## 1. Introduction

Uterine artery embolization (UAE) is an effective hemostatic treatment for postpartum hemorrhage (PPH) to avoid hysterectomy. Although hemostasis is achieved in over 90% of the cases, uterine necrosis occurs in 1.4% to 2.7% of the patients after UAE [1–3], and hysterectomy is required in nearly all the cases with necrosis [4].

Previous studies have reported that the size and type of embolic agents as well as maternal conditions, such as obesity, caesarean section, and placental abruption, are related to uterine necrosis after UAE [4, 5]. Preeclampsia (PE) is characterized by systemic vascular endothelial dysfunction that leads to enhanced vascular permeability, resulting in intravascular volume depletion and ascites. Ascites is independently associated with adverse maternal outcomes in patients with PE [6]. Patients with PE often present with disseminated intravascular coagulation (DIC) after delivery that can result in PPH [7]. However, the association between uterine necrosis after UAE and PE is not clarified [5]. Here, we report a case of uterine necrosis after UAE for PPH in a patient with PE associated with massive ascites.

## 2. Case Report

The patient was a 30-year-old, G1P0 woman. The body mass index (BMI) was 18.6 before conception. Her pregnant course was uneventful after natural conception. At 38 weeks and 4 days of gestation, she was hospitalized due to premature rupture of the membrane (PROM). After admission, she was diagnosed with PE since her blood pressure was 146/105 mmHg and urine protein-to-creatinine ratio was 629 mg/gCr. There was no cardiomegaly or pleural effusion in chest radiograph.

After her cervix was dilated with a metreurynter balloon, labor was induced with oxytocin. However, the labor did not progress on the day of admission or the following day. Labor induction with oxytocin was continued for 2 days after PROM while the patient's blood pressure was maintained around 140/90 mmHg with continuous drip infusion of nicardipine. The labor progressed after onset, and the transabdominal ultrasonography showed small volume of ascites. After 3 hours of the second stage of labor, the baby was delivered with forceps due to nonreassuring fetal status. The neonate weighed 2466 g with an Apgar score of 8/9, and umbilical artery pH was 7.159.

**Figure 1:**
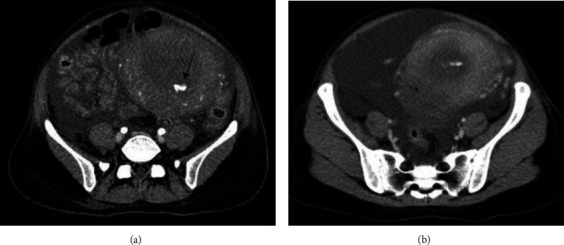
(a) Contrast-enhanced computed tomography images at the time of massive postpartum hemorrhage: extravasation from the uterine cavity (→). (b) massive ascites.

**Figure 2:**
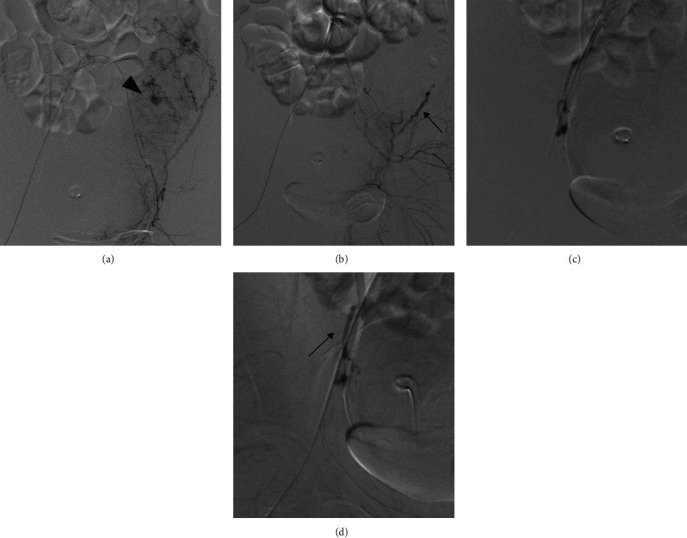
(a, b) The images of the left uterine artery before and after embolization. The extravasation (►) is no longer seen after embolization. The flow in the left uterine artery is preserved after embolization (→). (c, d) The images of the right uterine artery before and after embolization. The extravasation is not detected in the right uterine artery. The flow in the right uterine artery is also preserved after embolization (→).

The total blood loss was 800 g within 2 hours after delivery, but the hemorrhage increased to a total of 1,500 g 3 hours after delivery. Her heart rate was 100−110 beats per minute, and blood pressure was 110−120/60−80 mmHg. Her hemoglobin decreased to 6.0 g/dl, and fibrinogen was 199 mg/dl. We continuously administered oxytocin, inserted an intrauterine balloon inflated with 200 ml of distilled water, and administered blood transfusion: 2 units of mannitol-adenine-phosphate (MAP), 2 units of fresh frozen plasma (FFP), and 12 units of cryoprecipitate. Her blood pressure persisted over 110/60, and the shock index did not exceed 1 despite the hemorrhage. Contrast-enhanced computed tomography (CT) showed extravasation within the uterus, along with massive ascites (Figure 1). We decided to perform UAE for the hemostasis, and the extravasation from the left uterine artery was confirmed during the procedure. Using 1−2 mm gelatin sponge, we embolized bilateral uterine arteries. The flow from bilateral uterine arteries to the uterus was preserved after the embolization (Figure 2). The vital sign had been stable, and the shock index was constantly below 1 during UAE. The total blood loss was 2,070 g to the end of artery embolization, and the total amount of blood transfusion was 10 units of MAP, 8 units of FFP, and 12 units of cryoprecipitate.

**Figure 3:**
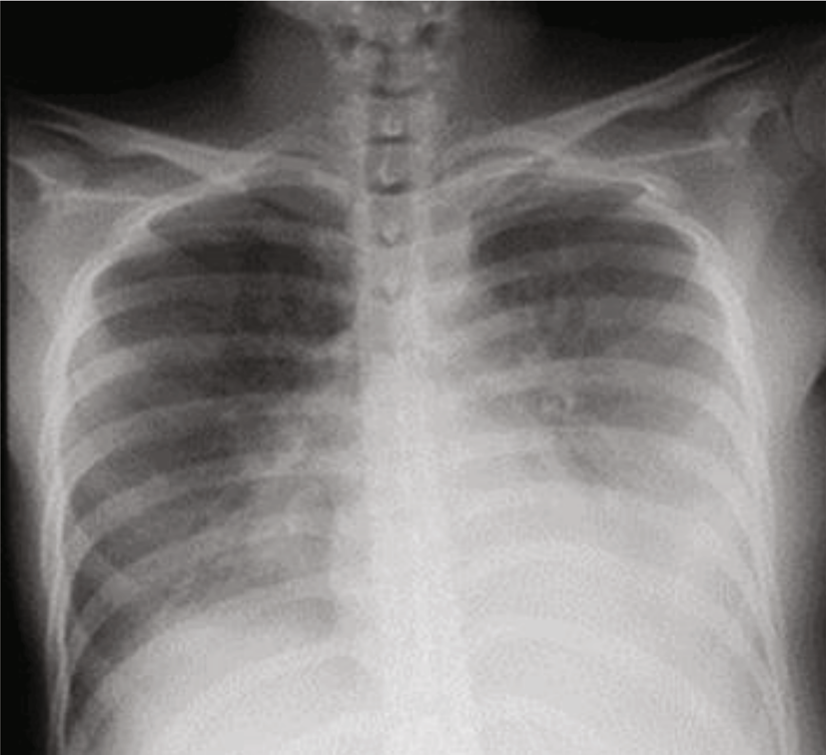
Chest radiograph at postpartum day 2 shows bilateral pleural effusion.

Although UAE enabled hemostasis, ascites resulted in abdominal distention, and pulmonary edema deteriorated (Figure 3). Continuous oxygen supplementation had been continued. Echocardiography did not demonstrate any abnormality, and the B-type natriuretic peptide (BNP) level was within normal. The continuous nicardipine drip also had been restarted after the procedure to control the blood pressure under 140/90. The blood oxygen saturation (SpO_2_) had been kept over 95%. On postpartum day 2, she was given oral antihypertensives to replace continuous nicardipine drip. We removed 2,000 ml of ascites by paracentesis and administered albumin 75 g/day because oral intake was difficult and marked respiratory distress occurred. Just after ascites removal, the patient did not demonstrate any drop in blood pressure or tachycardia. We initiated continuous administration of loop diuretics 84 mg/day until postpartum day 4 while tapering the amount due to oliguria. We paid attention to inferior vena cava diameter during diuretic administration, but it never showed any collapse. Hematocrit level, urea nitrogen/creatinine ratio, and electrolyte levels were steady. Her blood pressure and SpO_2_ had been stable after the ascites removal. The oliguria was improved from postpartum day 3. On postpartum day 6, oxygen administration and the continuous nicardipine drip were stopped. Pleural effusion and ascites ameliorated on postpartum day 7, and she did not need any diuretics. The proteinuria improved from postpartum day 10.

**Figure 4:**
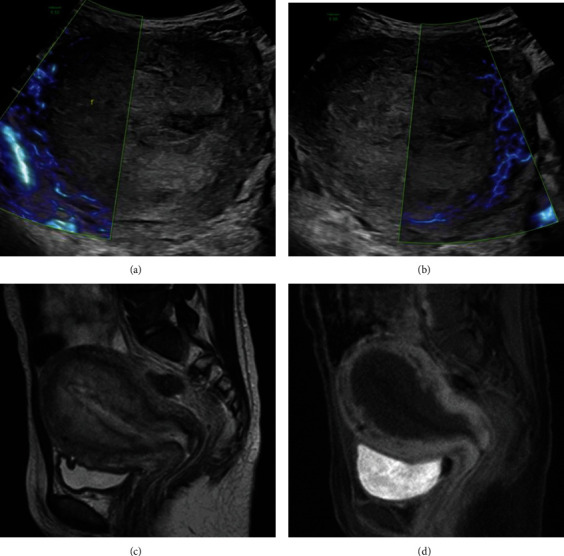
(a, b) Cross section of the internal myometrium in abdominal ultrasonography on postpartum day 24. Abdominal ultrasonography does not show blood flow in the internal myometrium by abdominal ultrasound. (c, d) MRI shows heterogeneous high intensity in internal myometrium on sagittal T2-weighted images (c) without contrast enhancement on sagittal postcontrast T1-weighted images (d), indicating necrosis.

However, tachycardia, elevated inflammatory response, and spike fever over 38 degrees had continued since postpartum day 2, and *Escherichia coli* was detected from lochia culture. Although the antibiotic was changed from clindamycin to tazobactam and piperacillin on postpartum day 6, then switched to meropenem on day 19, the patient's spike fever persisted. On postpartum day 24, we performed abdominal ultrasonography, using 3D convex probe (Voluson E10 BT19; GE Healthcare, Tokyo, Japan). The power Doppler sonography with slow flow mode, which can depict slow blood flow and microvascular blood flow with high sensitivity, detected no blood flow in the internal myometrium of the uterus. Magnetic resonance imaging (MRI) demonstrated a heterogeneous high-intensity area in the internal myometrium on T2-weighted images without contrast enhancement on postcontrast T1-weighted images (Figure 4). We diagnosed the patient with uterine necrosis, and we performed laparoscopic hysterectomy.

**Figure 5:**
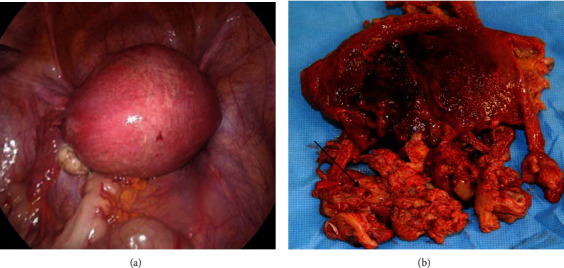
(a) Laparoscopic images of the uterine surface during surgery. The uterine surface appeared normal. (b) Macroscopic finding of the resected uterus shows the inner myometrium of the uterus is necrotized (→).

Although the surface of the uterus appeared to be normal, the resected internal myometrium was reddish-black and malodorous (Figure 5). Pathology confirmed myometrial necrosis with infiltration of massive inflammatory cells, such as neutrophils, lymphocytes, and plasma cells. The postoperative course was uneventful. She stopped oral antihypertensives on postpartum day 24. She was discharged on postoperative day 6 (postpartum day 30).

## 3. Discussion

Complications of UAE for obstetric hemorrhage such as intrauterine infection or pelvic abscess are reported to occur in 6%−7% of cases [1]. Uterine necrosis has been reported to occur in 1.4% to 2.7% following UAE [2, 3]. Factors that may associate with uterine necrosis include the size and type of the embolic agent in UAE [4]. Temporary embolic agents < 500 *μ*m cause narrow vessels to be embolized, which may lead to organ necrosis [1, 4]. We used 1–2 mm gelatin sponge, a temporary embolic agent, which is presumed to have little association with uterine necrosis.

The present case developed uterine necrosis after UAE for PPH and required hysterectomy. There is only one case report of uterine necrosis after UAE in a patient with PE by Hirashima et al. [8]. Pleural effusion, proteinuria, and atonic bleeding were observed in our case as well as the case reported by Hirashima et al. In addition, our case showed massive ascites. In PE, systemic vascular endothelial dysfunction is known to trigger various organ damage. Ascites in PE is mainly caused by hypoproteinemia and enhanced vascular permeability [6, 9]. Although transabdominal ultrasonography prior to labor showed a small volume of ascites, it suddenly increased after delivery. It suggests that severe vascular endothelial dysfunction occurred and caused systemic hypoperfusion associated with local circulatory failure in the organs, the mechanism by which the uterus may have become more vulnerable to necrosis. PE with ascites should be considered as a risky condition for uterine necrosis after UAE.

To inspect the pathophysiology mentioned above, we reviewed the past cases in our institution. We experienced 45 postpartum hemorrhage cases which required UAE for hemostasis in the past decade from January 2011 to December 2020. The present case was the first case of uterine necrosis after UAE for PPH. Among 45 cases, 10 cases were complicated with PE. We compared the present case with other PE cases (*n* = 9) (Table 1). Pulmonary edema was observed in 5 cases including the present case. The circulatory disturbance and hemorrhagic shock were confirmed in other cases, but not in this case. Diuretics were used in other PE cases. However, massive ascites was confirmed only in this case. These results support the suggestion that PE with massive ascites may be at high risk for uterine necrosis after UAE. This study design has been approved by our institutional ethics committee.

In addition, hemorrhagic shock and sepsis have been indicated as risk factors for uterine necrosis after UAE [4]. This patient had complicated premature rupture of membranes before birth comparing other PE cases which needed UAE. This patient also took more than 2 days until delivery and had fever prior to labor with an elevation of inflammatory response, all of which indicating a latent infection localized in the uterus. The association of uterine necrosis with these events, which have potentially exacerbated localized circulatory disturbance, cannot be ruled out.

On the other hand, we performed paracentesis and administered diuretics to control the massive ascites. Furosemide, a loop diuretic, activates the renin-angiotensin system, the vasopressin system, catecholamine, and others, potentially triggering electrolyte imbalances and renal dysfunction; therefore, it should be administered with caution in patients with PE [10, 11]. However, we consider that systemic circulatory disturbance was unlikely to be elicited by these procedures. This is because we kept monitoring the inferior vena cava diameter using ultrasonography while simultaneously administering albumin. The patient did not demonstrate inferior vena cava collapse, renal dysfunction, or any other signs of intravascular volume depletion. And there were three other cases of PE which needed diuretics after UAE without uterine necrosis in our cases.

The characteristic symptoms of uterine necrosis are fever, abdominal pain, pelvic pain, sepsis, metrorrhagia, and vaginal discharge [4]. In our patient, only fever persisted despite the use of multiple antimicrobial agents. Blood flow was confirmed only in the external layer in the uterine myometrium, and no blood flow in the internal layer was observed by ultrasonography. Contrast-enhanced MRI showed loss of contrast enhancement in the internal myometrium, leading us to diagnose uterine necrosis. These imaging findings are characteristic for uterine necrosis [8, 12]. In the event of fever of unknown origin or other persistent symptoms indicative of uterine necrosis after UAE, observing the state of the endometrium with abdominal ultrasonography is important for diagnosis. When hypoperfusion in the internal myometrium is observed by ultrasonography, contrast-enhanced MRI should be considered for definitive diagnosis of uterine necrosis.

In conclusion, patients with PE with massive ascites may possess a greater risk of UAE-induced uterine necrosis due to uterine local circulatory failure associated with intravascular volume depletion. In UAE for patients with PE, care should be taken in the selection of embolic materials and the management of other risk factors such as infection or hypovolemic status for uterine necrosis. After UAE, it is important to pay attention to the symptoms suspecting uterine necrosis, as well as evaluate the vascularity of the uterine with ultrasonography and MRI to achieve timely diagnosis of uterine necrosis.

## Figures and Tables

**Table 1:** The characteristics of PE cases which required uterine artery embolization after delivery in our hospital from January 2011 to December 2020. Massive ascites before delivery is confirmed only in the present case (No. 1). Gelatin sponge is used as embolic agent in all patients except for No. 10.

	Ascites	Pulmonary edema	Diuretics	Embolic agent	Embolization of uterine arteries	Shock index > 1	Transfusion
No. 1	++	+	+	Gelatin sponge	Bilateral	—	+
2	—	—	—	Gelatin sponge	Bilateral	—	+
3	—	—	—	Gelatin sponge	Bilateral	+	+
4	—	+	+	Gelatin sponge	Bilateral	+	+
5	—	+	—	Gelatin sponge	Bilateral	+	+
6	—	+	—	Gelatin sponge	Bilateral	+	+
7	—	+	+	Gelatin sponge	Unilateral	+	+
8	—	—	—	Gelatin sponge	Unilateral	+	+
9	—	+	—	Gelatin sponge	Bilateral	—	+
10	—	+	+	NBCA^∗^	Unilateral	+	+

^∗^NBCA: n-butyl-2-cyanoacrylate.
